# Evaluation of classical and deep-learning deformable registration for magnetic resonance to four-dimensional computed tomography contour mapping in liver stereotactic body radiotherapy

**DOI:** 10.1016/j.phro.2026.100969

**Published:** 2026-04-17

**Authors:** Ziad Kheil, Laurent Risser, Elizabeth Cohen-Jonathan Moyal, Soleakhena Ken

**Affiliations:** a Univ Toulouse, Oncopole Claudius Regaud, IUCT-Oncopole, CRCT Radopt Team, UMR 1037 INSERM, Toulouse, France; b Univ Toulouse, Oncopole Claudius Regaud, IUCT-Oncopole, Physics Department, Toulouse, France; c Univ Toulouse, Paul Sabatier, Toulouse, France; d Univ Toulouse, Institut de Mathématiques de Toulouse, CNRS, Toulouse, France

**Keywords:** DIR, Multi-modal treatment planning, Deep learning

## Abstract

**Background and purpose:**

: In liver stereotactic body radiotherapy (SBRT), magnetic resonance imaging (MRI)-to-four-dimensional computed tomography (4D-CT) contour mapping remains challenging because of cross-modality differences and respiratory motion, motivating fast automated solutions. We comparatively evaluated the clinical practicality of deep learning (DL) based deformable image registration (DIR).

**Materials and Methods:**

: This retrospective study included 4D-CT and 3D MRI scans from 170 patients treated with liver SBRT. Segmentations were obtained using open-source automated tools, and test-set labels were clinician reviewed. The dataset was split at the patient level into training/validation/testing sets (122/32/16), an additional 5-fold cross-validation was performed on the training+validation cohort. We compared three approaches: an optimization-based B-spline DIR tool (NiftyReg), a single-network DL model (Direct), and a two-step DL pipeline decomposing multi-modal alignment and intra-4D-CT motion tracking. Statistical analysis used patient-level paired Wilcoxon signed-rank tests; organ-wise secondary analyses were corrected using the Benjamini–Hochberg false discovery rate (FDR; q=0.05).

**Results:**

Classical B-spline DIR achieved the best all-label averages (Dice 0.71 vs 0.62; 95th percentile Hausdorff distance [H95], 17.7 mm vs 21.0 mm). DL achieved its strongest performance on supervised structures compared to all labels (e.g., Dice 0.71 vs 0.62; H95 18.9 mm vs 21.0 mm) while reducing runtime from ∼59 s to 0.09 s per registration. The two-step pipeline improved deformation regularity (foldings 0.009% vs 0.025% for Direct).

**Conclusion:**

DL pipelines provided competitive contour-mapping accuracy with orders-of-magnitude runtime gains, but performance depended strongly on the choice of training supervision; classical DIR remained strongest on global all-label metrics.

## Highlights

• NiftyReg achieved the best global organ alignment (Dice 0.71 vs 0.62).
• Deep learning performed best on supervised organs (e.g. liver Dice 0.86 vs 0.84).
• Separating registration steps reduced foldings (0.009% vs 0.025%).
• Deep-learning performance depended strongly on supervision choices.

## Graphical abstract

**Figure:**
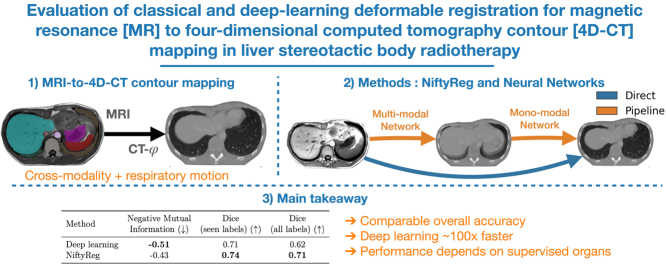

## 1 Introduction

Stereotactic Body Radiation Therapy (SBRT) planning routinely relies on 4D-CT to account for respiratory motion and on accurate delineation of targets and organs-at-risk [1]. MRI is frequently acquired to improve soft-tissue visualization, but clinical use requires robust contour mapping from MRI to the planning 4D-CT phases. This remains challenging due to cross-modality appearance differences, respiratory motion, and partial field-of-view overlap [2].

Deformable image registration (DIR) is widely studied for contour mapping and related RT tasks. However, international surveys and clinical reviews have reported heterogeneous adoption and persistent barriers to the routine use of multimodal DIR, notably quality assurance (QA), uncertainty management, and workflow integration [2], [3], [4]. A 2019 international survey found that only 19% of clinics used DIR for CT–MRI alignment [3], and recent data from Swiss centers reported increasing availability (61%) but still limited routine use (33%) [5].

Compared with classical optimization-based DIR [6], [7], [8], [9], prior work showed that learning-based methods could amortize registration to enable fast inference once trained [10], and could explicitly leverage available contours during training to encourage contour-aligned deformations.

Despite growing interest, clinically robust MRI/4D-CT registration remains elusive. Some centers have relied on non validated, in-house solutions [4], lacking standardization. Methods often applied MRI/CT registration directly to the 4D-CT setting without considering the added mobility component, which resulted in suboptimal deformation fields due to conflicting optimization goals. This is compounded by DIR’s sensitivity to task-specific losses and hyperparameters.

Mono-modal 4D-CT registration has been well studied, with public datasets and benchmarks supporting both optimization and deep learning approaches [11], [12], [13], [14], [15]. Yet these efforts primarily addressed CT-to-CT phase alignment. In parallel, multimodal DIR methods (e.g., CT–MRI) typically targeted a single reference CT phase. To our knowledge, the combined problem—mapping MRI-defined contours to *any* 4D-CT phase—has not been systematically evaluated, despite being central for leveraging MRI soft-tissue contrast in liver SBRT planning.

To address this implementation gap, we performed a pragmatic comparative evaluation of MRI-to-4D-CT contour mapping under respiratory motion using clinically realistic supervision. We investigated whether deep learning could provide a clinically viable solution to the combined problem of MRI-to-4D-CT alignment and intra-4D-CT motion tracking. To this end, we synthesized the existing literature into a DIR pipeline and applied it to a real-world clinical dataset of 170 patients, with annotations from open-source software [16]. We benchmarked our method against standard DIR approaches and observed major runtime gains (sub-second per registration) while maintaining competitive contour agreement overall, with the strongest performance on structures explicitly supervised during training. We also assessed whether addressing motion-tracking and multi-modal DIR jointly or separately yielded better results. This approach produced encouraging results on real-world data using only freely available annotations.

## 2 Materials and methods

### 2.1 Understanding recent advances in DIR

We benchmarked deep learning image registration (DLIR) against classical iterative DIR and compared two DL designs: single-network (Direct) vs. two-step (Pipeline).

The goal of DIR is to estimate a dense deformation field $\Phi_{M\to F}$ mapping a moving image M to a fixed image F. In radiotherapy, classical DIR is typically formulated as an optimization balancing image similarity, regularization, and task-specific constraints [17].

To fix notation, we write a standard DLIR objective used to combine image similarity, regularization, and optional task-specific terms [17], [18]:

(1) $$\Phi_{M\to F}^{*}=\underset{\Phi }{arg min}L_{sim}\left(F,M\;\circ \;\Phi \right)+\lambda L_{reg}\left(\Phi \right)+\gamma L_{additional}$$

with λ, γ∈R being weight coefficients associated to the different functions.

Despite rapid progress, many DNN-based DIR methods are trained and evaluated on curated datasets that under-represent clinical variability [19]. Performance can therefore be sensitive to distribution shift and may require re-tuning outside the training domain [19]. Deployment is further limited by scarce annotations, artifacts, variable fields-of-view (FoV), and brittle preprocessing (e.g., rigid misalignment).

### 2.2 Contour mapping pipeline design

For this study, it was of interest to investigate a real-world dataset in order to examine how to bridge the gap between research and clinical application. Throughout the following, we denote a deformation field mapping the moving image M to the fixed image F as $\Phi_{M\to F}$. Furthermore, we specify the imaging modality through the notations MR for the MRI modality described in Section 2.6 and CT-φ for a CT scan at phase φ∈T=[0,16%,33%,50%,66%,83%].

Our registration pipeline for contour mapping required overcoming misalignment due to abdominal organ movements on one hand, and intrinsic differences in acquisition modalities. We proposed to remedy these problems one by one by composing the resulting registration fields. In practice, this facilitated training two separate neural networks, on the different registration objectives. Hence, the pipeline consisted of an initial rigid pre-registration step followed by two DNNs: $g_{\theta_{multi}}$, which performed multi-modal registration from MRI to CT-50, and $f_{\theta_{mono}}$, which performed mono-modal temporal registration between CT phases.

Once the networks were trained, we used $g_{\theta_{multi}^{*}}$ to infer the deformation field $\Phi_{\text{MR}\to \text{CT-50}}$ mapping the MRI to the expiration-phase CT. We then applied $f_{\theta_{mono}^{*}}$ to compute the five fields ${\left(\Phi_{\text{CT-50}\to \text{CT-}\varphi }\right)}_{\varphi \in T}$ capturing respiratory motion. The MRI segmentation map $S_{\text{MR}}$ was then propagated to each 4D-CT phase by composing these fields. This dichotomy enabled us to train two independent networks, each optimized for its specific task without compromising on training objectives:

(2) $$\Phi_{\text{MR}\to \text{CT-}\varphi }=\Phi_{\text{CT-50}\to \text{CT-}\varphi }\;\circ \;\Phi_{\text{MR}\to \text{CT-50}}=g_{\theta_{multi}^{*}}\left(\text{MR},\text{CT-50}\right)\;\circ \;f_{\theta_{mono}^{*}}\left(\text{CT-50},\text{CT-}\varphi \right)$$

### 2.3 Model architectures

We used a standard, well-established backbone (Attention U-Net [20]) as a strong baseline, and focused instead on adapting the problem formulation and evaluation to real world radiotherapy constraints (artifacts in imaging, differences in field-of-view, automated annotation). The ablation study examining hyper-parameter choices is discussed in Supplementary material A, and quantitative results for different network backbones and regularization approaches are shown in Figures S1–S2.

Input images were stacked channel-wise to produce a (2,H,W,D) image which was used by the network to produce a stationary velocity field. The use of velocity fields allowed to obtain near-diffeomorphic deformation fields, and was experimentally validated in Section 2.6. The predicted stationary velocity field (SVF) was integrated over 7 time steps to produce a dense displacement field (DDF), which warped the moving image into the final output using a Spatial Transformer Network (STN) [21].

Distinct training objectives were applied to the two models each matched to their respective sub-tasks.

### 2.4 Temporal model

In the mono-modal, temporal branch, images share similar intensity distributions, allowing the network to focus purely on organ motion across respiratory phases. This enabled training with a Local Normalized Cross-Correlation (LNCC) loss, which was expected to offer improved stability over mutual information–based alternatives in this setting [22], [23], [24], and better capture large, physiologically plausible deformations.

Given the complex motions involved—like lung expansion or liver sliding—regularization was key to producing smooth, realistic deformations. Additionally, 4D-CT artifacts could introduce local discontinuities [25], further complicating registration as illustrated in Supplementary material B (Figures S3–S5). This motivated a task-specific loss that avoided penalizing large but plausible deformations, while discouraging foldings or extreme volume changes by constraining deviations in the determinant of the jacobian of Φ to the identity matrix: $L_{DetJac}=\frac{1}{H\times W\times D}\sum {\left(\left|\nabla \Phi \right|-1\right)}^{2}$ where ∇Φ is the jacobian of the deformation field and |⋅| the determinant operator [26].

An auxiliary Dice loss enforced a soft constraint on physically acceptable solutions giving the following learning objective:

(3) $$\theta_{mono}^{*}=\underset{\theta }{arg min}L_{LNCC}\left(\text{CT-}\varphi ,\text{CT-50}\;\circ \;f_{\theta }\left(\text{CT-50},\text{CT-}\varphi \right)\right)+\lambda L_{DetJac}\times \left(f_{\theta }\left(\text{CT-50},\text{CT-}\varphi \right)\right)+\gamma L_{Dice}\left(S_{\text{CT-}\varphi },S_{\text{CT-50}}\;\circ \;f_{\theta }\left(S_{\text{CT-50}},S_{\text{CT-}\varphi }\right)\right)\;,$$

with $f_{\theta }$ the mono-modal registration network parametrized by θ.

### 2.5 Multi-modal training

The multi-modal branch began with rigid alignment, needed due to differing resolutions, shapes, and fields of view across modalities. We resampled and zero-padded the MRI to match the CT-50 reference, then optimized rigid parameters to coarsely align expiration-phase images.

Given MRI/CT FoV mismatch, we restricted all computations to their overlap. After resampling MRI to CT-50 spacing and zero-padding, we retained the native MRI support mask; following rigid expiration-phase pre-alignment, we mapped this mask to CT space to define $\Omega_{\cap }$. Multi-modal similarity and metrics for partially covered structures were computed only within $\Omega_{\cap }$ (see Supplementary material C and Figures S6–S7 for dataset construction, preprocessing details and examples).

Despite similar organ positioning at expiration, visual discrepancies between modalities complicated alignment. To guide this, we minimized the negative mutual information (−MI) as the similarity loss (equivalently maximizing MI; lower is better), supplemented by an auxiliary Dice loss. A first-order regularization on deformation magnitudes ensured only small, plausible warps were introduced:

(4) $$\theta_{multi}^{*}=\underset{\theta }{arg min}L_{MI}\left(\text{CT-50},\text{MR}\;\circ \;g_{\theta }\left(\text{MR},\text{CT-50}\right)\right)+\lambda {\left|\left|\nabla g_{\theta }\left(\text{MR},\text{CT-50}\right)\right|\right|}_{2}+\gamma L_{Dice}\left(S_{\text{CT-50}},S_{\text{MR}}\;\circ \;g_{\theta }\left(S_{\text{MR}},S_{\text{CT-50}}\right)\right)\;,$$

where $g_{\theta }$ is the multi-modal registration network, with parameters θ.

### 2.6 Experimental protocol

**Fig. 1:**
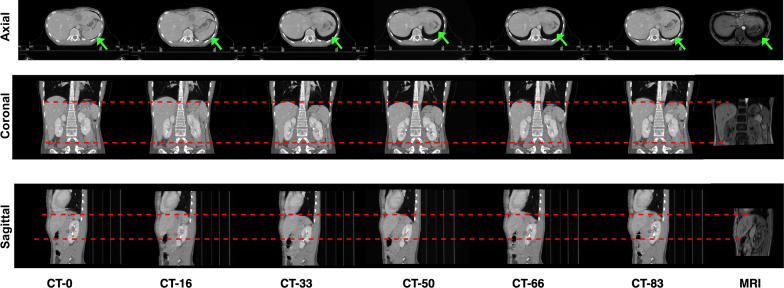
Coronal, axial and sagittal views of a patient’s 4D-CT volumes over the respiratory cycle (6 phases) and companion auxiliary breath-held MRI on expiration (50%). Notice the large respiratory motion across CT phases, as well as contrast differences and field-of-view discordance between CT and MRI.

To the best of our knowledge, no public MRI/4D-CT registration dataset existed. We therefore assembled an in-house clinical cohort^1^ of 170 patients with 4D-CT and MRI acquisitions, as illustrated in Fig. 1. All patients underwent SBRT for liver tumors; although the target site is abdominal, planning scans often included partial thoracic coverage, enabling reporting of additional held-out organ metrics. Further details on the dataset and preprocessing are provided in Supplementary material C. Patients were split at the patient level into training (122), validation (32), and test (16) subsets for both mono- and multi-modal experiments (Table 1).

Organ masks were generated using TotalSegmentator [16]. The following structures were segmented: liver, spleen, left and right kidneys, vertebrae, lungs, heart, stomach, pancreas, and bowels. For training, we supervised four abdominal organs (liver, spleen, left and right kidneys) using a Dice loss, as these were consistently visible across CT and MRI and provided stable supervision. A quality control (QC) filter removed failed automatic segmentations (Dice below a predefined threshold on a labeled subset) to avoid biasing training and evaluation. A senior medical physicist reviewed all test-set annotations and confirmed anatomical consistency; the corresponding sensitivity analysis is reported in Supplementary material D, and sample segmentations are displayed in Figure S7. For evaluation, all available structures were used to provide a broader clinical assessment and to probe generalization beyond the supervised organs.

At test time, all evaluated methods were tasked with aligning ${\left(\mathrm{MRI},\mathrm{CT}\text{-}\varphi \right)}_{\varphi \in T}$ pairs from the held-out cohort (16 patients; 96 pairs). Deformation fields were predicted/optimized at low resolution (128,128,64) and bilinearly upsampled to the original image size (512,512,D). In addition to this fixed test set, we performed 5-fold cross-validation with patient-level splitting on the training+validation cohort for the DL methods only (see Table S3); NiftyReg was not cross-validated due to runtime constraints.

**Fig. 2:**
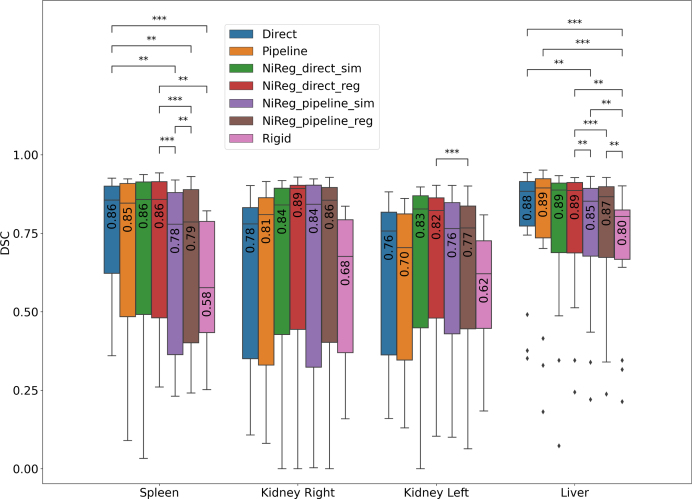
Box plots of Dice scores after registration for the organs used during training across the investigated methods. (ns: 0.05<p≤1, *: 0.01<p≤0.05, **: 0.001<p≤0.01, ***: $10^{-4}<p\leq 0.001$, ****: $p\leq 10^{-4}$).

Following recent DIR QA recommendations [4], we reported complementary metrics. Structural alignment was assessed using the Dice similarity coefficient (DSC) and the 95th percentile Hausdorff distance (H95), reported separately for supervised (training) organs and held-out structures to quantify generalization. Loss and evaluation were restricted to overlapping regions: supervision was applied only to organs fully contained in the FoV, and per-organ metrics were computed for organs fully and partially located in the common FoV. Deformation plausibility was quantified via the percentage of folding voxels (Foldings) and the standard deviation of the log-Jacobian determinant (StdLJ), computed both globally and within organ masks (Foldings_mask, StdLJ_mask). Finally, mutual information (MI) was reported between fixed and warped images globally and within the overlap region. For statistical comparisons, we applied paired Wilcoxon signed-rank tests to patient-level median values, with Benjamini–Hochberg false discovery rate correction (FDR; q = 0.05) applied across the set of pairwise comparisons displayed in Fig. 2; only significant FDR-adjusted comparisons are annotated.

### 2.7 Implementation details

As a baseline, we applied a single rigid registration (*Rigid*) to align the MRI to the CT reference phase (CT-50, end-expiration). This reflected current clinical workflows, where MR–CT pairs are often rigidly aligned in treatment planning systems (TPS). We then evaluated NiftyReg [27], a widely used B-spline DIR tool. While NiftyReg is a research implementation, its transformation models (rigid and free-form B-spline deformation) mirror those embedded in many TPS platforms (e.g., MIM, Velocity), where rigid registration is clinically validated and B-spline–based DIR is commonly provided as an option [2]. NiftyReg was evaluated in four configurations: two were used to directly register MR images to any 4D-CT volume, while two others were employed in a pipeline fashion similarly to our DL pipeline: MR → CT-50 → CT-φ. In both cases, we tested two sets of hyper-parameters: one focused on optimizing similarity metrics (*NiReg_sim*) and another with stronger regularization (*NiReg_reg*). Hyper-parameters for both were tuned on three validation samples due to the high computational cost, with full implementation details provided in Supplementary material E.

A total of three DNNs were evaluated on the test set. The first one, *Direct*, was trained to register MRI images to any CT-φ image from the corresponding patient. The two others were used jointly to evaluate the pipeline described in Section 2.2 (*Pipeline*). In all cases, DNN hyper-parameters were determined through a comprehensive grid-search on the validation set. Models with the best validation performance were retained. Model-specific hyper-parameters are provided in Table 1, while the training and grid-search procedures are described in Supplementary material A.

**Table 1:** Hyper-parameters used for final model training.

Direct	Multi-modal	λ	γ	Loss	Training Set
		0.5	0.5	Eq. (4)	366 (MR/CT-φ)
Pipeline	Multi-Modal	0.5	0.5	Eq. (4)	122 (MR/CT-50)
	Temporal (Mono-Modal)	0.3	0.7	Eq. (3)	400 (CT-50/CT-φ)

## 3 Results

We found a clear trade-off between deep learning and classical DIR. While NiftyReg achieved the best global all-label averages (DSC 0.708, H95 17.718mm), the DL-based approaches still achieved good overall alignment and performed best on structures explicitly supervised during training, reaching a DSC of 0.711±0.229 on these structures (Table 3). In the overlapping FoV region, the DNN methods achieved the best similarity of all methods (−MI=−0.510±0.072 for *Direct*). Moreover, DL approaches displayed a striking speed advantage: sub-second inference on GPU, meanwhile optimizing a single displacement field with NiftyReg required over 2 min (12 min for the complete 4D-CT in Table 2). On the other hand, Table 2 further showed that while DL models still produced some folding voxels (less than 0.1% of the DDF), NiftyReg produced very regular fields, fully satisfying diffeomorphic properties due to the inherent regularity of the underlying B-spline approach.

Performance depended strongly on the organ and on whether it was used for supervision during training. For example, DL outperformed NiftyReg on liver alignment (DSC 0.86 vs. 0.84), whereas the ranking reversed for the heart (DSC 0.70 vs. 0.78; Supplementary material F, Tables S1–S2). Across the four supervised organs, DL methods achieved median Dice scores between 0.70 and 0.86, with statistically significant differences from several NiftyReg variants on spleen and liver (Fig. 2). The full set of organ-wise boxplots, including H95, organs not used during training, and annotated significant FDR-adjusted comparisons, is shown in Supplementary Figure S8.

Both DL approaches yielded very similar similarity and alignment metrics, with differences of 0.01 in overlapping-region −MI, 0.01 in all-label DSC, and 0.3 mm in all-label H95 (Table 3). The main advantage of the two-step pipeline was improved deformation plausibility: decomposing the warp into two simpler sub-tasks yielded smoother fields (StdLJ 0.37 vs. 0.39) and fewer folding voxels (0.009% vs. 0.025%; Table 2).

Patient-level 5-fold cross-validation confirmed the same trends as the held-out test set, as shown in Supplementary material G (Table S3). Both DL designs improved over the rigid baseline, increasing seen-label Dice from 0.71 to 0.81 and unseen Dice from 0.63 to 0.70. Performance remained better on supervised than unseen organs (DSC: 0.81 vs. 0.70), while alignment differences between *Direct* and *Pipeline* were small.

**Fig. 3:**
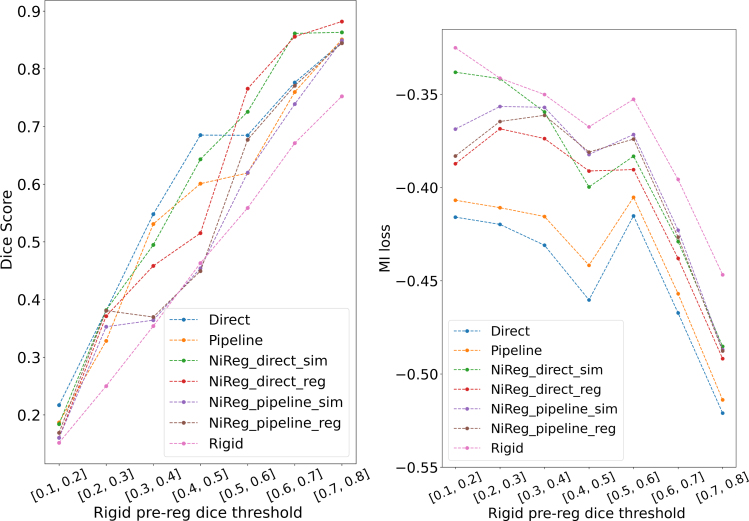
Evolution of average Dice scores (left) and average mutual information similarity (right) after the evaluated deformable registration methods (y-axis), as a function of the Dice score after initial rigid pre-registration, grouped into bins (x-axis). Each bin represents the range of initial rigid Dice scores, illustrating how DIR improves alignment depending on the quality of the rigid pre-registration.

Fig. 3 shows Dice scores and mutual information after DIR as a function of the rigid pre-registration Dice score. The relative order of the methods was maintained across bins, and all deformable approaches improved Dice by approximately 0.2 over rigid registration, indicating that rigid registration alone was insufficient in this setting.

Although the improvements were larger when the initial rigid pre-registration was of better quality, DIR generally still improved the results even in cases with poor initial alignment. For example, in the 0.4–0.5 rigid-registration Dice bin, the best NiftyReg approach reached only about 0.5 Dice after DIR, whereas the DL methods reached about 0.6–0.7. In contrast, in the 0.7–0.8 bin, where rigid alignment was already relatively good, NiftyReg improved Dice to nearly 0.9, whereas the DL methods reached about 0.83. In addition, some configurations of NiftyReg occasionally deteriorated mask alignment relative to the rigid baseline in some bins, whereas DL-based methods showed more consistent improvement across the full range of rigid-registration quality (Fig. 3). This pattern suggests greater robustness of the DL models to variations in initial alignment, whereas the fixed NiftyReg hyper-parameters may have been suboptimal in some cases.

**Table 2:** Mean ± standard deviation of metrics pertaining to deformation-field regularity and plausibility. Folding values are percentages of total displacements considered. Bold values are best, italics are second best.

Method	Full Field		FoV		Time (s) (↓)
	Foldings (%) (↓)	StdLJ (↓)	Foldings_Mask (%) (↓)	StdLJ_mask (↓)	
Direct	0.025 ± 0.023	0.39 ± 0.07	0.055 ± 0.086	0.89 ± 0.26	**0.094 ± 0.004**
Pipeline	0.009 ± 0.010	0.37 ± 0.04	0.039 ± 0.069	0.86 ± 0.17	*0.15 ± 0.11*
NiReg_direct_sim	**0.000 ± 0.000**	0.30 ± 0.12	**0.000 ± 0.000**	0.29 ± 0.12	58.6 ± 19.9
NiReg_direct_reg	**0.000 ± 0.000**	0.13 ± 0.13	**0.000 ± 0.000**	0.18 ± 0.19	59.2 ± 19.9
NiReg_pipeline_sim	**0.000 ± 0.000**	*0.12 ± 0.06*	**0.000 ± 0.002**	*0.12 ± 0.06*	127.6 ± 24.5
NiReg_pipeline_reg	**0.001 ± 0.003**	**0.06 ± 0.02**	*0.018 ± 0.059*	**0.06 ± 0.02**	126.1 ± 21.9

**Table 3:** Mean ± standard deviation of similarity and mask-alignment metrics. Bold values are best, italics are second best. For MI, we report −MI (negative mutual information), hence lower is better.

	Method	MI (↓)		DSC (↑)		H95 (↓)	
		Whole image	Overlapping region	Seen labels	All labels	Seen labels	All labels
Rigid	Rigid	−0.23 ± 0.05	−0.44 ± 0.06	0.62 ± 0.22	0.60 ± 0.23	21.2 ± 15.0	22.1 ± 17.8
DL	Direct	−0.29 ± 0.06	**−0.51 ± 0.07**	*0.71 ± 0.23*	0.62 ± 0.25	*18.9 ± 16.5*	21.0 ± 19.4
	Pipeline	−0.29 ± 0.06	*−0.50 ± 0.07*	0.69 ± 0.25	0.61 ± 0.26	19.2 ± 17.2	21.3 ± 21.4
NiftyReg	NiReg_direct_sim	**−0.42 ± 0.12**	−0.43 ± 0.08	0.71 ± 0.28	*0.68 ± 0.26*	19.8 ± 20.9	*19.3 ± 21.7*
	NiReg_direct_reg	*−0.32 ± 0.10*	−0.43 ± 0.08	**0.74 ± 0.25**	**0.71 ± 0.24**	**18.0 ± 18.7**	**17.7 ± 20.2**
	NiReg_pipeline_sim	−0.27 ± 0.06	−0.42 ± 0.08	0.68 ± 0.26	0.66 ± 0.25	20.4 ± 17.5	19.9 ± 19.4
	NiReg_pipeline_reg	−0.29 ± 0.06	−0.42 ± 0.08	0.69 ± 0.26	0.67 ± 0.25	20.4 ± 18.7	19.7 ± 20.4

## 4 Discussion

Our findings suggest that deep learning-based DIR can substantially improve workflow efficiency for MRI-to-4D-CT contour mapping, but that accuracy depends strongly on the supervision used during training. In particular, the contrast between supervised and held-out organs indicates that faster inference alone is not sufficient for clinical deployment; the choice of training supervision, especially in weakly supervised settings, and evaluation breadth remain critical [19]. Our work underscores label selection as an important clinical design decision for DLIR deployment. The observed limitations of rigid pre-alignment further support the need for deformable approaches in respiratory settings, consistent with prior reports that rigid transforms cannot capture motion-induced deformation in 4D imaging [28], [29].

Overall, this study provided a pragmatic RT-oriented evaluation of MRI-to-4D-CT contour mapping in liver SBRT using widely available open-source components and QA-relevant endpoints [30], including runtime, contour agreement, image similarity, and deformation plausibility. The proposed learning-based approaches amortize the cost of classical DIR’s per-case hyperparameter tuning across a training population, offering a path toward standardized, operator-independent contour propagation in liver SBRT [31]. Although the present results do not yet establish clinical readiness, they emphasize that supervision strategy and data coverage are central to reliable DL deployment [19]. The sub-second inference demonstrated here is also directly relevant to online adaptive RT, where contour propagation must occur within treatment session time constraints [32]. Alignment quality on supervised structures was furthermore in a range compatible with typical inter-observer contouring variability in abdominal RT [33], suggesting clinical potential for well-represented organs.

These findings should be interpreted in light of several limitations. Training supervision relied on automated contours, although all test-set labels were clinician reviewed. NiftyReg was evaluated only on the fixed held-out test set, without cross-validation, due to computational cost. Severe 4D-CT reconstruction artifacts, such as those caused by irregular breathing, introduced local discontinuities that challenged motion estimation [34]. Although dose recalculation was outside the scope of this study, such artifacts may affect downstream dose mapping and warrant dedicated QA when DIR is used for dose-related applications. Comparison with commercial TPS-integrated dose mapping was also limited because deformation fields were typically not accessible from these systems.

Future work should investigate end-to-end training of the two-step pipeline, which may reduce compounding errors between the multi-modal and temporal components. This was not feasible here because jointly loading, training, and back-propagating through both models exceeded the available computational resources. Additional work should also examine these methods on broader multi-center datasets with richer annotations.

## CRediT authorship contribution statement

**Ziad Kheil:** Writing – review & editing, Writing – original draft, Visualization, Validation, Supervision, Software, Resources, Project administration, Methodology, Investigation, Funding acquisition, Formal analysis, Data curation, Conceptualization. **Laurent Risser:** Writing – review & editing, Writing – original draft, Visualization, Validation, Supervision, Software, Resources, Project administration, Methodology, Investigation, Funding acquisition, Formal analysis, Data curation, Conceptualization. **Elizabeth Cohen-Jonathan Moyal:** Supervision. **Soleakhena Ken:** Writing – review & editing, Writing – original draft, Visualization, Validation, Supervision, Software, Resources, Project administration, Methodology, Investigation, Funding acquisition, Formal analysis, Data curation, Conceptualization.

## Funding

This work was supported by a MESRI (Ministère de l’Enseignement supérieur, de la Recherche et de l’Innovation) doctoral grant from the French Ministry of Higher Education, Research and Innovation .

## Declaration of competing interest

The authors declare the following financial interests/personal relationships which may be considered as potential competing interests: The last co-author of this manuscript Elizabeth Moyal Cohen-Jonathan reported research funding from AstraZeneca, Novocure, Bayer, and Incyte. Scientific advisory board member and intellectual property with Novocure.

Other authors declare no conflicts of interest.

## Appendix A Supplementary data

MMC S1 Supplementary data including ablation studies, visual registration samples, and organ-specific performance metrics’. MMC S1

## Footnotes

1 The study received institutional review board approval and is registered under code F20220824152603.
