# Supplementary material for: Evaluation of classical and deep-learning deformable registration for magnetic resonance to four-dimensional computed tomography contour mapping in liver stereotactic body radiotherapy
Source: Phys Imaging Radiat Oncol. 2026 Apr 17;39:100969. doi: 10.1016/j.phro.2026.100969 (PMC13129378; doi:10.1016/j.phro.2026.100969)
Supplement: MMC S1 — Supplementary data including ablation studies, visual registration samples, and organ-specific performance metrics’. [file mmc1.pdf]

Supplementary material A. Ablation results

Training: The models were trained over 400 epochs with a batch size of 10 pairs of images. They were optimized using the Adam optimizer with an initial learning rate of $1e^{-3}$, and a halving of the learning rate when the validation plateaued for 40 epochs. The networks predicted a velocity field which was integrated using the MONAI library over 7 time steps to produce dense displacement fields,

Hyper-parameters:A comprehensive grid search was carried out to identify the most suitable hyper-parameters for our task, with a focus on both network architecture and training regularization. A wide range of hyper-parameters related to the neural networks and their training procedures were investigated. The mono-modal and multi-modal networks had the same hyper-parameter search space, mainly: the type of similarity loss and regularization penalty used, the weights of regularization and Dice terms in the loss, the backbone neural architecture, and the use of velocity fields.

Notably, model architecture had a significant impact on performance, as shown by the validation losses (similarity, Dice, and regularization) in Figure S1 (smoothed using exponential moving averages for clarity). As discussed in Section 2.5, regularization penalties affected not only the smoothness and plausibility of the predicted deformation fields but also mask alignment and similarity metrics, as depicted in Figure S2.

These ablation results highlight the importance of carefully selecting both architectural and training hyper-parameters to ensure reliable and accurate registration.

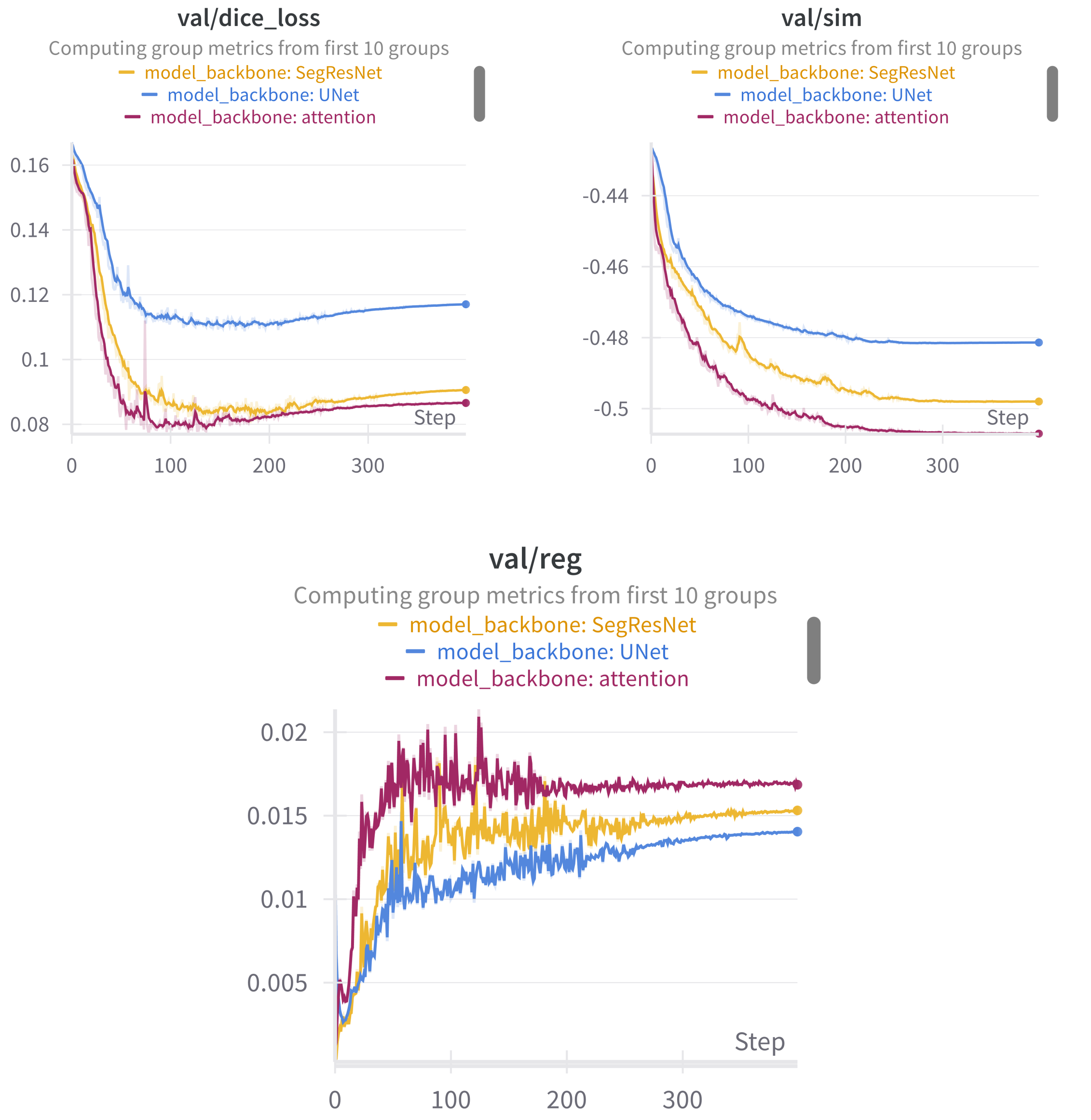

Figure S1: Effects of model architecture: Top left (Dice), Top right (Sim), Bottom (Reg).

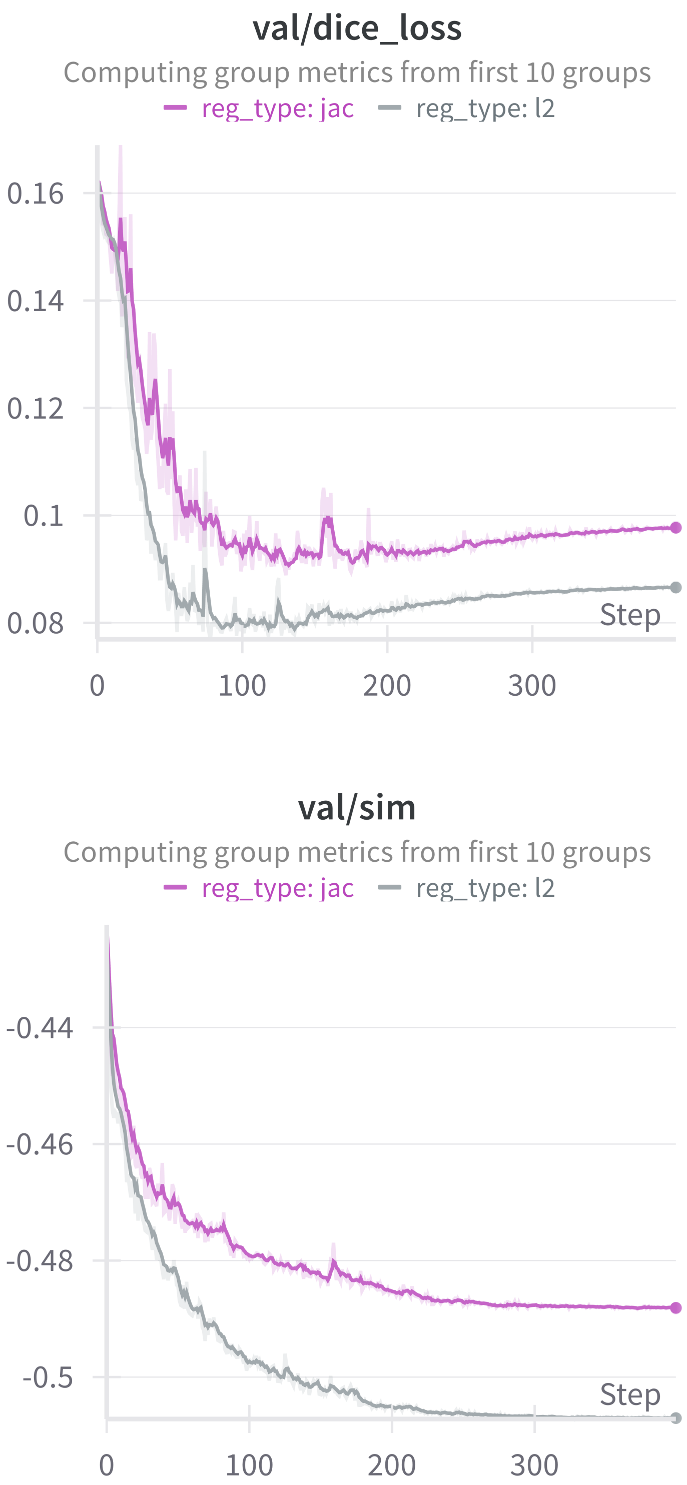

Figure S2: Averaged effects of differing regularization penalty type of the multi-modal network on the Dice and Similarity validation loss during training. Results were averaged over other varying parameters. Jacobian regularization seems to lead to poorer alignment metrics, but is essential in order to reduce the foldings found in the predicted deformation field.

Supplementary material B. Sample registrations

Three samples form the test set with different rigid pre-registration results were deformably registered to showcase the methods. Figures S3, S4 and S5 display the axial, coronal and sagittal views of the images. The figures contain the fixed CT reference, the moving MRI, and the warped MRI using three methods, alongside the deformation fields at the slice of interest. We also display the contour of the liver automatic segmentation as well as the deformed contour in red.

Note that Sample S5 displays a faulty rigid pre-registration which impacts all methods, but severely limits the NiftyReg approach.

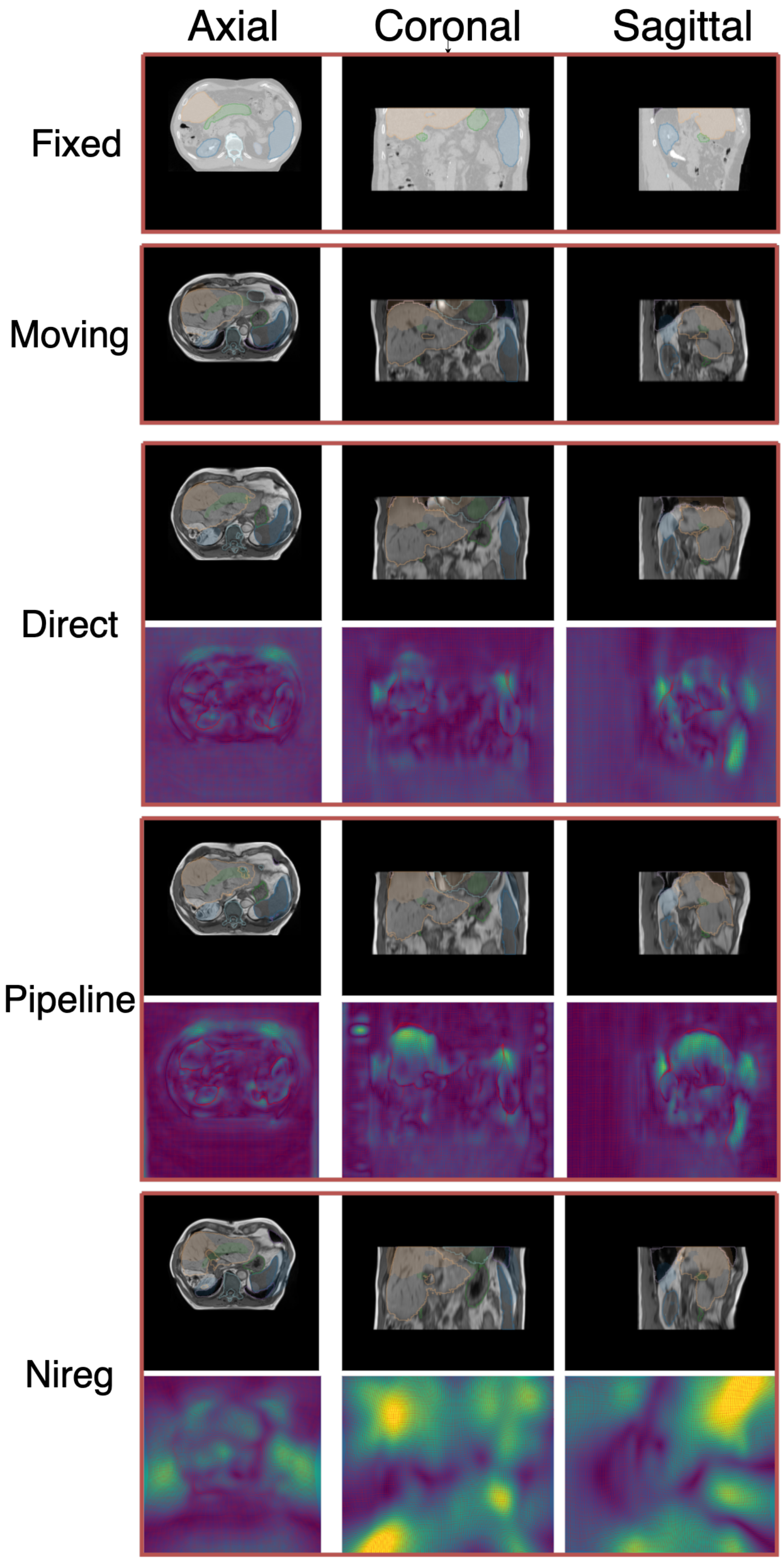

Figure S3: Patient 138 registration between MRI and CT-0.33.

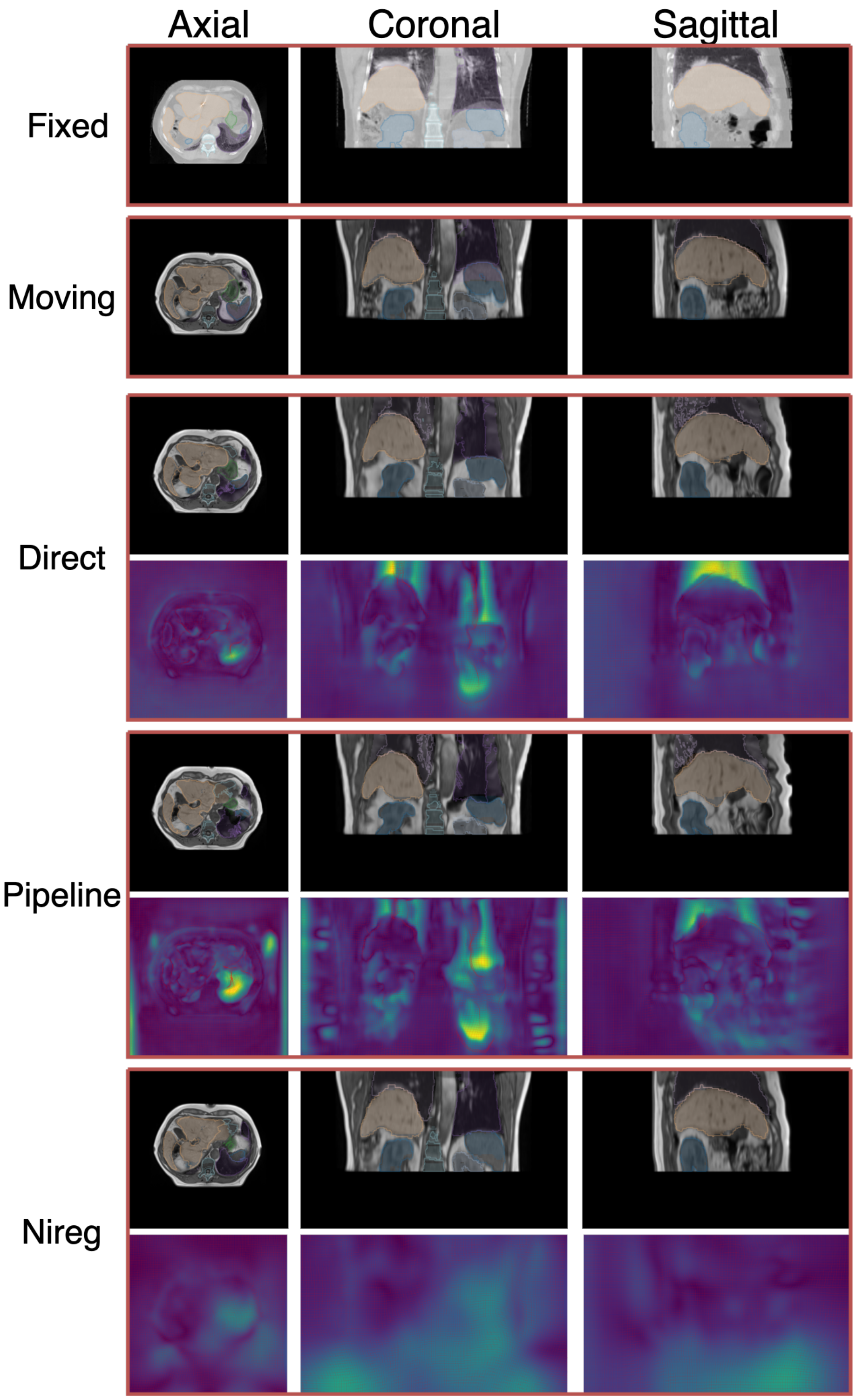

Figure S4: Patient 158 registration between MRI and CT-0.66.

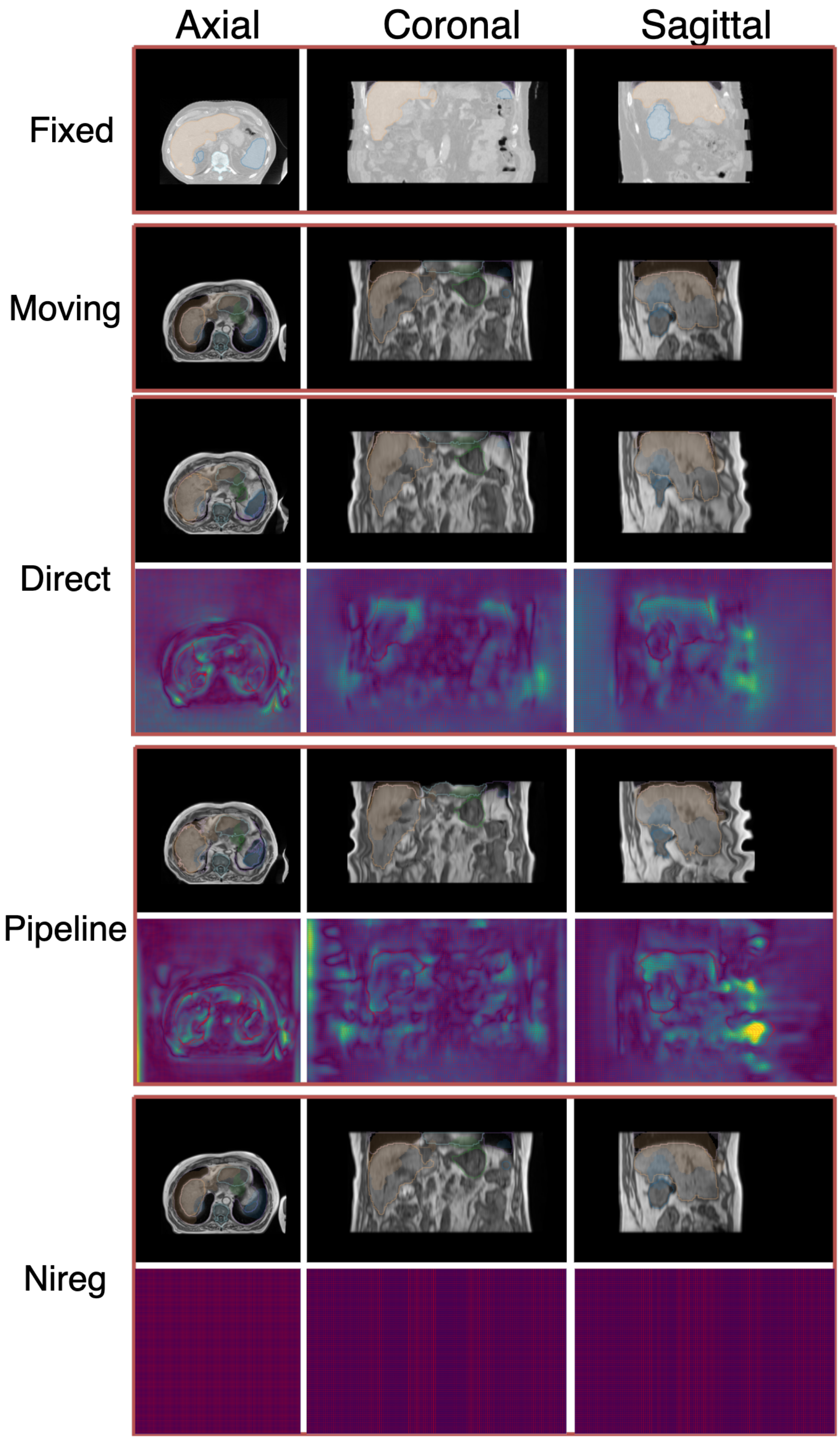

Figure S5: Patient 109 registration between MRI and CT-0.0.

Supplementary material C. Dataset construction

MRI consisted in a rapid 3D axial acquisition with patient compatible volumetric interpolated breath-hold examination with two-point water-fat separation. They were acquired on a Magnetom Aera 1.5T (Siemens, Erlangen, Germany)

4D-CT images were acquired as contiguous stack of axial slices with audio-coaching instructions or in free-breathing mode and reconstructed as 3D volumes ordered in six phases representative of the patient's respiratory cycle. This was done through an Optima RT32 scanner (General Electric, Waukesha, WI)

Pre-processing: A standard pre-processing pipeline for abdominal CT and MR images was used. CT scan values were clipped to the (-1000HU,400 HU) range then min-max normalized to the [0,1]. During training and validation the images were resized to the fixed shape (128,128,64) and the pixel spacing was left as is (anisotropic). MR images were resampled to match CT voxel spacing, cropped and padded to obtain the same shape, and then rigidly registered to CT-50 before training. This single rigid alignment step ensures comparability across modalities, though it does not compensate for respiratory motion. They were then normalized to the [0,1] range. Finally, masks corresponding to roughly matching regions of interest in the MRI and CT scans were automatically extracted, and used during training to mask the deformation field before back-propagation.

In order to train and validate the two networks, a multi-modal dataset comprised of (MRI,CT-50) was extracted from the previous patient sets. In the mono-modal case, for a given patient's 4D-CT, pairs of (fixed,moving) images were constructed by randomly pairing two CT scans at different breathing phases. This gives a total of 1830 (training), 480 (validation) and 240 (test) distinct pairs. We randomly subsampled 400 pairs from the training set and 50 validation pairs to keep training and validation computing times tractable, and conserved the test set unmodified.

The complete pre-processing outline is displayed in Figure S6

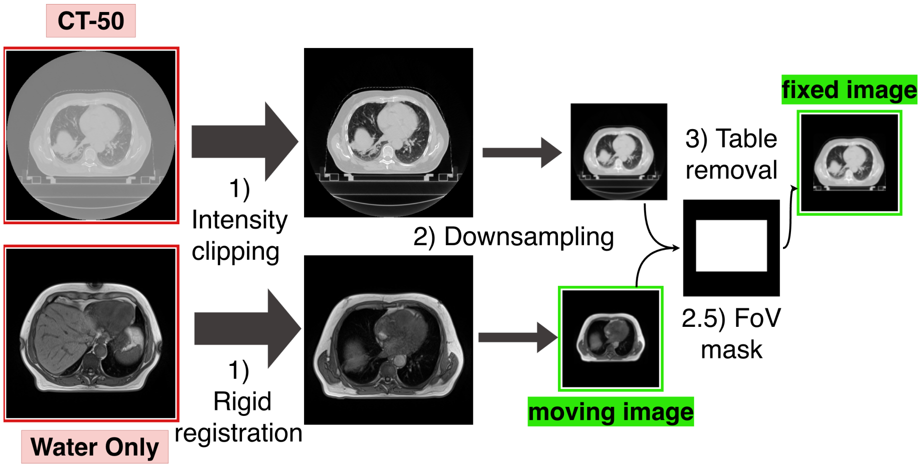

Figure S6: Outline of the image preprocessig pipeline.

Supplementary material D. Sensitivity of DSC to minor contour truncations

Segmentations obtained using the automated tool were reviewed by an expert physicist. Minor under-segmentation was occasionally observed (typically 1–2 axial slices near the diaphragmatic dome or peripheral lung), with < 2 percentage-point impact on DSC in a sensitivity analysis.

To estimate the effect these, we simulated slice omissions/additions on representative test-set masks computed DSC differences. Across affected organs, the absolute DSC change remained below 0.02 (i.e., < 2 percentage points), indicating that such small contour differences do not materially affect the conclusions.

Furthermore, in Figure S6 we display sample segmentation masks obtained using the automated tool for different phases of a 4D-CT volume, as well as the associated MRI. We can see certain breathing artifacts and how they were handled by the annotation tool.

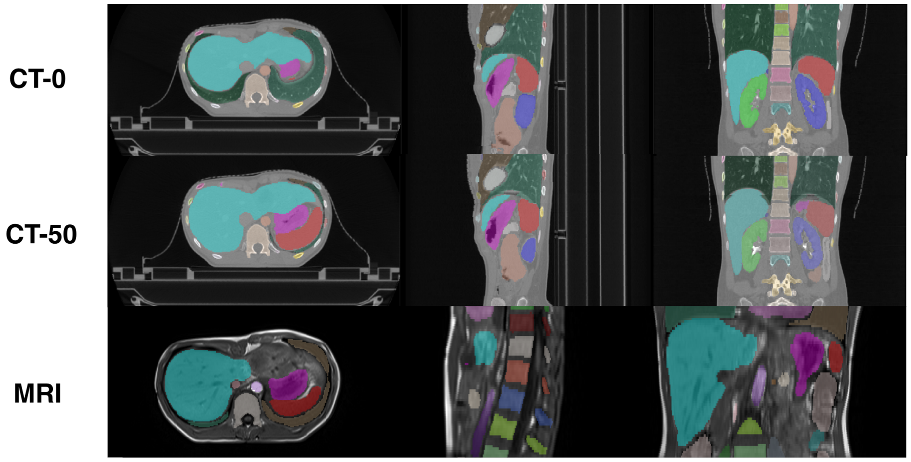

Figure S7: Sample segmentations for two volumes of a 4D-CT and an MRI.

Supplementary material E. Software and hardware

Deep learning experiments and metric computation were performed in Python using MONAI (`monai==1.4.0`). Automatic organ segmentations were obtained with TotalSegmentator (`TotalSegmentator==2.7.0`). Statistical comparisons and significance annotations were performed with `statannotations` (`statannotations==0.6.0`). Classical DIR baselines used NiftyReg [1]. All experiments were run on an NVIDIA GeForce RTX 3090 GPU.

The NiftyReg package offers a vast number of hyper-parameters which can be used to tune the registration algorithm. We fixed these hyper-parameters by comparing results on 3 random validation set patients. The search space included: similarity loss, regularization loss and weights, use of velocity fields, use of pyramidal approach, number of level to perform.

Both NiftyReg metrics were computed using mostly default parameters. Non default ones were: using velocity field integration approach (*-vel*), no pyramidal approach (*-nopy*), and Jacobian determinant penalty term (*-jl*). For *NiReg_reg* the jacobian term was weighted by $1e^{-2}$, and $1e^{-3}$ for *NiReg_sim*.

[1] `https://github.com/KCL-BMEIS/niftyreg`

Supplementary material F. Detailed results

Tables S1 and S2 give a detailed overview of Dice values and H95 distances for the segmented organs.

	Spleen	Kid. R	Kid. L	Liver	Stom.	Panc.	Lung L	Lung R	Bowel	Vert.	Heart	Avg
Rigid	0.64 ± 0.20	0.64 ± 0.19	0.61 ± 0.16	0.77 ± 0.14	0.46 ± 0.23	0.39 ± 0.24	0.68 ± 0.21	0.71 ± 0.21	0.38 ± 0.13	0.62 ± 0.08	0.67 ± 0.24	0.60 ± 0.23
Direct	**0.81 ± 0.14**	0.70 ± 0.21	0.68 ± 0.20	**0.86 ± 0.12**	0.50 ± 0.26	0.39 ± 0.26	0.62 ± 0.19	0.65 ± 0.17	0.32 ± 0.14	0.64 ± 0.07	0.70 ± 0.24	0.62 ± 0.25
Pipeline	0.78 ± 0.19	0.71 ± 0.23	0.64 ± 0.22	*0.85 ± 0.15*	0.50 ± 0.28	0.40 ± 0.27	0.60 ± 0.21	0.63 ± 0.22	0.32 ± 0.14	0.64 ± 0.07	0.64 ± 0.28	0.61 ± 0.26
NiReg_direct_sim	0.78 ± 0.23	*0.76 ± 0.23*	*0.71 ± 0.24*	0.84 ± 0.14	*0.61 ± 0.28*	*0.49 ± 0.27*	*0.74 ± 0.24*	*0.77 ± 0.24*	0.38 ± 0.16	0.68 ± 0.08	0.73 ± 0.24	0.68 ± 0.26
NiReg_direct_reg	*0.80 ± 0.21*	**0.79 ± 0.22**	**0.75 ± 0.20**	0.84 ± 0.14	**0.62 ± 0.28**	**0.50 ± 0.25**	**0.77 ± 0.22**	**0.80 ± 0.22**	**0.45 ± 0.15**	0.70 ± 0.09	**0.78 ± 0.24**	0.71 ± 0.24
NiReg_pipeline_sim	0.71 ± 0.24	0.72 ± 0.25	0.69 ± 0.21	0.81 ± 0.14	0.58 ± 0.27	0.47 ± 0.26	0.70 ± 0.25	0.75 ± 0.22	0.42 ± 0.15	**0.71 ± 0.10**	0.75 ± 0.27	0.66 ± 0.25
NiReg_pipeline_reg	0.72 ± 0.23	0.75 ± 0.22	0.69 ± 0.21	0.82 ± 0.14	0.59 ± 0.28	0.46 ± 0.25	0.70 ± 0.26	0.76 ± 0.22	*0.44 ± 0.15*	*0.70 ± 0.10*	*0.75 ± 0.26*	0.67 ± 0.25

Table S1: Detailed Dice Scores (higher is better) per organ. Kid: Kidney, Stom: Stomach, Panc: Pancreas, Vert: Vertebrae. Last column is the global average.

	Spleen	Kid. R	Kid. L	Liver	Stom.	Panc.	Lung L	Lung R	Bowel	Vert.	Heart	Avg
Rigid	21.2 ± 11.3	14.0 ± 11.1	14.8 ± 10.5	21.8 ± 11.9	28.5 ± 15.7	19.3 ± 11.7	22.8 ± 9.4	24.5 ± 15.5	**49.7 ± 30.8**	8.7 ± 2.1	17.5 ± 12.9	22.1 ± 17.8
Direct	14.3 ± 13.0	13.6 ± 11.1	14.9 ± 12.7	**17.9 ± 13.6**	26.3 ± 16.5	19.9 ± 13.5	20.3 ± 5.8	20.2 ± 7.0	56.0 ± 35.1	8.4 ± 2.0	18.4 ± 13.0	21.0 ± 19.4
Pipeline	15.1 ± 13.7	12.5 ± 11.0	15.4 ± 12.8	18.6 ± 15.2	27.0 ± 17.9	20.3 ± 14.4	20.0 ± 6.6	18.8 ± 9.5	58.4 ± 41.0	8.6 ± 2.3	18.9 ± 15.1	21.3 ± 21.4
NiReg˙direct˙sim	*14.0 ± 14.7*	12.7 ± 14.4	*14.0 ± 15.6*	18.4 ± 13.9	25.6 ± 20.9	*18.1 ± 16.1*	*16.9 ± 15.6*	*14.8 ± 10.5*	54.9 ± 39.3	7.8 ± 2.3	14.7 ± 12.1	19.2 ± 21.7
NiReg˙direct˙reg	**13.8 ± 13.4**	**9.7 ± 11.4**	**12.6 ± 12.3**	*18.0 ± 14.0*	**23.9 ± 19.7**	**17.3 ± 13.1**	**13.5 ± 8.8**	**12.7 ± 9.1**	53.6 ± 36.9	*7.0 ± 2.0*	**12.8 ± 12.5**	17.7 ± 20.2
NiReg˙pipeline˙sim	17.3 ± 12.8	12.5 ± 12.6	15.1 ± 13.7	20.8 ± 13.2	25.6 ± 19.1	19.1 ± 14.1	18.6 ± 9.5	17.8 ± 10.9	*51.7 ± 34.0*	**6.9 ± 2.1**	13.4 ± 13.3	19.9 ± 19.4
NiReg˙pipeline˙reg	17.1 ± 12.7	*11.3 ± 11.6*	15.8 ± 13.8	19.9 ± 14.6	*25.4 ± 19.9*	19.6 ± 14.5	17.7 ± 9.7	16.1 ± 10.1	53.6 ± 37.7	7.1 ± 2.1	*13.1 ± 11.8*	19.7 ± 20.4

Table S2: Detailed H95 distance (lower is better) per organ. Kid: Kidney, Stom: Stomach, Panc: Pancreas, Vert: Vertebrae. Last column is the global average.

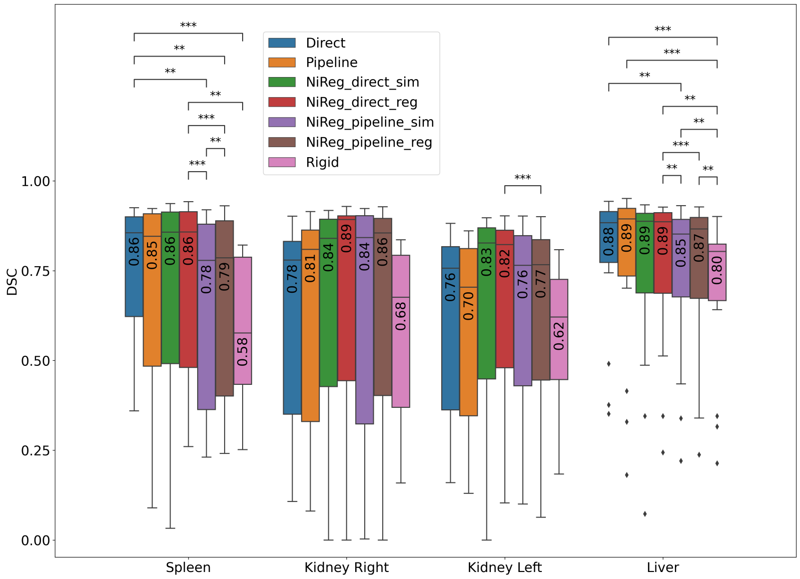

(a) Dice Scores: Organs included during training (Supervised).

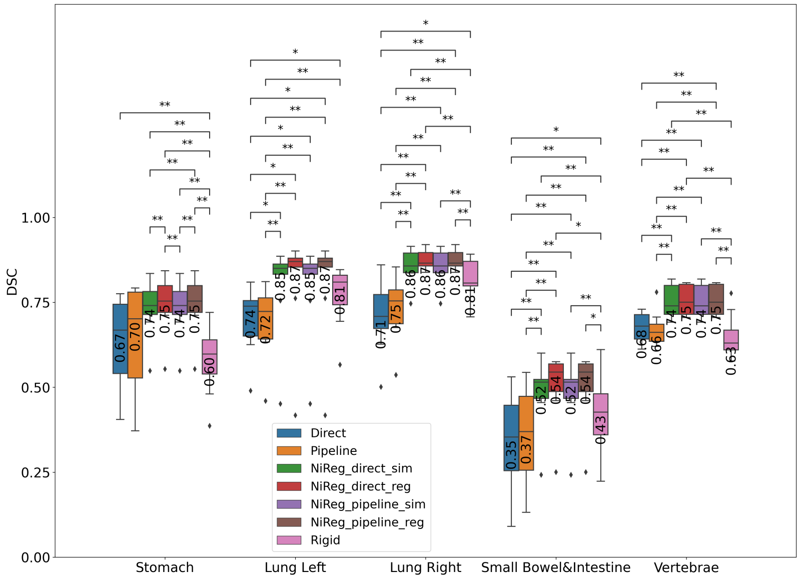

(b) Dice Scores: Organs not used in training (Unseen).

Figure S8: Detailed performance metrics for investigated methods (Part I). Top sections (a-b) display DICE scores. (Continued on next page)

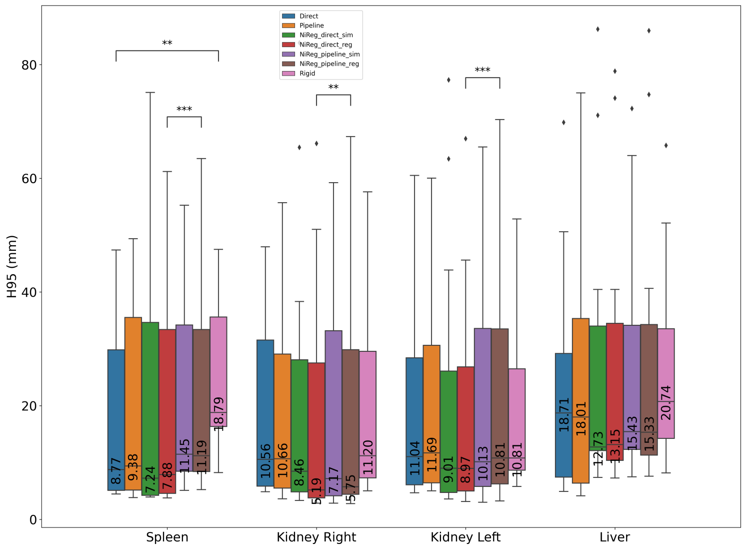

(c) H95 Distance: Organs included during training (Supervised).

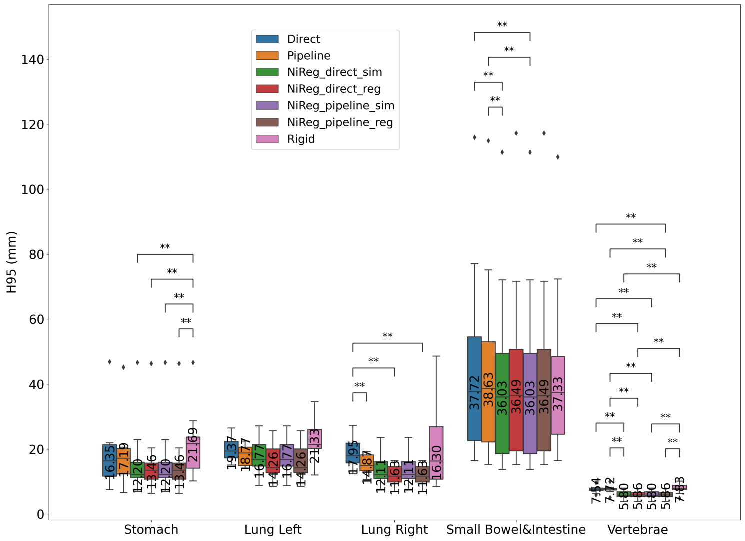

(d) H95 Distance: Organs not used in training (Unseen).

Figure S8: Detailed performance metrics for investigated methods (Part II). Top sections (a-b) display DICE scores, while bottom sections (c-d) display Hausdorff 95th percentile (H95) values. Sections (a, c) show supervised organs; sections (b, d) show unsupervised organs. In order to ensure a fair comparison, samples presenting faulty pre-processing steps were discarded from the analysis (e.g., rigid registration failures). Statistical comparisons were performed using paired Wilcoxon signed-rank tests on patient-level summaries, with Benjamini–Hochberg false discovery rate (FDR) correction applied over the set of comparisons displayed; only significant FDR-adjusted p-values are annotated (ns: $0.05 < p \leq 1$, *: $0.01 < p \leq 0.05$, **: $0.001 < p \leq 0.01$, ***: $10^{-4} < p \leq 0.001$, ****: $p \leq 10^{-4}$).

Supplementary material G. Cross-validation

To assess robustness to the train/validation split, we performed a five-fold cross-validation on the combined training+validation cohort. Splits were generated at the patient level (all phases from a given patient assigned to a single fold) to avoid leakage across correlated MR/CT-φ pairs; folds were randomly selected with patient-level stratification. For each fold, models were trained on four folds and evaluated on the held-out fold, and we report mean±SD within each held-out fold as well as mean±SD across fold means. The rigid baseline corresponds to the same rigid pre-alignment evaluated on the held-out fold prior to deformable registration.

	**Direct (DL)**				**Pipeline (DL)**				**Rigid baseline**			
	Seen		Unseen		Seen		Unseen		Seen		Unseen	
	Dice ↑	H95 ↓	Dice ↑	H95 ↓	Dice ↑	H95 ↓	Dice ↑	H95 ↓	Dice ↑	H95 ↓	Dice ↑	H95 ↓
Fold 1	0.81 ± 0.14	**13.4 ± 12.2**	**0.65 ± 0.21**	22.6 ± 25.3	**0.82 ± 0.13**	14.6 ± 13.1	0.65 ± 0.22	**21.9 ± 24.2**	0.73 ± 0.14	16.6 ± 11.5	0.58 ± 0.20	24.1 ± 20.0
Fold 2	0.83 ± 0.13	**10.6 ± 9.9**	**0.71 ± 0.20**	16.5 ± 17.1	**0.83 ± 0.13**	10.9 ± 10.2	**0.71 ± 0.21**	**16.3 ± 16.9**	0.74 ± 0.15	13.2 ± 8.6	0.65 ± 0.19	17.3 ± 13.1
Fold 3	**0.81 ± 0.13**	**12.0 ± 9.5**	**0.73 ± 0.20**	**14.6 ± 12.9**	0.80 ± 0.15	14.7 ± 12.7	0.72 ± 0.20	15.6 ± 13.7	0.71 ± 0.16	15.3 ± 9.0	0.64 ± 0.20	19.2 ± 14.3
Fold 4	**0.83 ± 0.13**	**11.7 ± 12.7**	**0.71 ± 0.20**	**16.6 ± 17.1**	0.82 ± 0.13	12.3 ± 11.7	0.70 ± 0.20	16.7 ± 17.0	0.74 ± 0.15	13.2 ± 8.6	0.65 ± 0.19	17.3 ± 13.1
Fold 5	0.76 ± 0.20	**12.1 ± 10.8**	**0.68 ± 0.23**	**18.0 ± 18.8**	**0.77 ± 0.19**	13.1 ± 13.7	0.68 ± 0.23	19.2 ± 23.5	0.65 ± 0.22	16.4 ± 11.6	0.61 ± 0.22	21.1 ± 16.2
Mean ± SD across folds	**0.81 ± 0.03**	**12.0 ± 1.0**	**0.60 ± 0.03**	**17.7 ± 3.0**	**0.81 ± 0.02**	13.1 ± 1.6	0.70 ± 0.03	17.9 ± 2.6	0.71 ± 0.04	14.9 ± 1.7	0.63 ± 0.03	19.8 ± 2.9

Table S3: Five-fold cross-validation on the training+validation cohort using patient-level splitting. Fold rows report mean ± SD within the held-out fold. The last row reports mean ± SD across folds (computed from the fold means). “Seen ” denotes organs used in the training loss; “Unseen” denotes organs evaluated without explicit supervision. The rigid baseline corresponds to the rigid pre-alignment prior to deformable registration.
